# Agronomic, economic, and environmental performance of nitrogen rates and source in Bangladesh’s coastal rice agroecosystems

**DOI:** 10.1016/j.fcr.2019.107567

**Published:** 2019-09-01

**Authors:** Shah-Al Emran, Timothy J. Krupnik, Virender Kumar, M. Yusuf Ali, Cameron M. Pittelkow

**Affiliations:** a Department of Crop Sciences, University of Illinois, Urbana, IL 61801, USA; b International Maize and Wheat Improvement Center (CIMMYT), Sustainable Intensification Program. House 10/B, Road 53, Gulshan-2, Dhaka, 1212, Bangladesh; c International Rice Research Institute (IRRI), DAPO Box 7777, Metro Manila 1301, Philippines; d Dhaka, Bangladesh

**Keywords:** Nitrogen use efficiency, Energy productivity, Greenhouse gas, Urea super granule (USG), Urea deep placement (UDP)

## Abstract

Farmers in low-elevation coastal zones in South Asia face numerous food security and environmental sustainability challenges. This study evaluated the effects of nitrogen (N) rate and source on the agronomic, economic, and environmental performance of transplanted and rainfed ‘*aman*’ (monsoon-season) rice in Bangladesh’s non-saline coastal areas. Fifty-one farmers participated in trials distributed across two landscape positions described as ‘highlands’ (on which field water inundation depth typically remains <30 cm) and ‘medium-highlands’ (inundation depths 30–90 cm) planted singly with varieties appropriate to each position (BRRI *dhan* 39 for highlands and the traditional variety *Bhushiara* for medium-highlands). Researcher designed but farmer-managed dispersed plots were located across three district sub-units (Barisal Sadar, Hizla, Mehendigonj) and compared N source (broadcast prilled urea or deep-placed urea super granules (USG)) at four N rates. Rice grown on medium-highlands did not respond to increasing N rates beyond 28 kg N ha^−1^, indicating that little fertilization is required to maintain yields and profitability while limiting environmental externalities. In highland locations, clear trade-offs between agronomic and environmental goals were observed. To increase yields and profits for BRRI *dhan* 39, 50 or 75 kg N ha^−1^ was often needed, although these rates were associated with declining energy and increasing greenhouse gas (GHG) efficiencies. Compared to prilled urea, USG had no impact on yield, economic, energy and GHG efficiencies in medium-highland locations. USG conversely led to 4.2–5.8% yield improvements at higher N rates on highlands, while also increasing energy efficiency. Given the observed yield, agronomic and economic benefit of USG, our preliminary results that farmers can consider use of USG at 50 kg N ha^−1^ to produce yields equivalent to 75 kg N ha^−1^ of prilled urea in highland landscapes, while also reducing environmental externalities. These results suggest that when assessing sustainable intensification (SI) strategies for rice in South Asia’s coastal zones, N requirements should be evaluated within specific production contexts (e.g. cultivar type within landscape position) to identify options for increasing yields without negatively influencing environmental and economic indicators. Similar studies in other parts of coastal South Asia could help policy-makers prioritize investments in agriculture with the aim of improving rice productivity while also considering income generation and environmental outcomes.

## Highlights

• Trials were conducted in highland and medium-highland coastal locations.
• On highlands, increasing inputs boosted yields and GHGs in HYV rice, and USG at 50 kg N ha^−1^ produced similar yield and economic efficiency as 75 kg N ha^−1^ prilled urea in highlands.
• Deep-placed urea did not generate higher profits on medium-highlands.
• Little N response occurred on medium-highlands with a traditional variety.
• This variety was associated with reduced energy requirements and GHG emissions.

## 1 Introduction

South Asia is home to more than one fifth of the world’s population, with estimates that 35% of its inhabitants live in coastal zones (Barbier, 2015). The region’s coastal populations are vulnerable to numerous environmental threats including cyclones, river bank erosion, salinity intrusion, water logging and flooding (Islam et al., 2011). With 711 km of coastline, Bangladesh is widely considered to be climate change vulnerable, while also being the world’s most densely populated country of notable land area (Hossain, 2011). And despite considerable progress in staple food production, Bangladesh continues to face important food security challenges due to increasing population, shifts in dietary preferences, lack of land availability for agricultural expansion, climatic production risks, and inequitable distribution of food (GFSI, 2017). Adding to these problems, prices for staple cereals have fluctuated widely in the last decade (FAO, 2018), with recent and noteworthy changes in rice in particular (USDA FAS, 2018).

Rice (*Oryza sativa*) is grown on close to 80% of Bangladesh’s arable land during three cropping seasons: *’Aus’* (the spring season, from March/April to June/July), *’Aman’* (the monsoon season, from June/July to September/October) and *’Boro’* (the winter season, from November/December to April/May) (BBS (Bangladesh Bureau of Statistics), 2016). Among the three seasons, *aman* comprises around 49% of the total rice cultivation area in Bangladesh (BBS (Bangladesh Bureau of Statistics), 2016). This crop is rainfed with average yields around 2.4 t ha^–1^, considerably lower than the 4.1 t ha^–1^ mean for other tropical rice producing countries in Asia (BBS, 2016; FAO, 2018). Increasing *aman* rice productivity is therefore important in efforts to boost food security, although agronomic research has tended to overlook cropping systems in environmentally challenging coastal areas. In these regions, the landscape position of farmers’ fields is an important factor influencing *aman* productivity. As a low-lying deltatic country, physical geographers and agronomists in Bangladesh classify ariable land types based on the average depth of water inundation experienced during the monsoon season. Major categories include ‘highlands’ (0–30 cm inundation depth), which comprise 30% of Bangladesh, ‘medium-highlands’ (30–90 cm inundation depth and 48% land area), ‘medium-lowlands’ (90–180 cm depth and 13% land area), ‘lowlands’ (8% land and 180–300 cm depth), and ‘very lowland’ (>300 cm depth, 1% area) (Brammer, 2012). Although this terminology can be counter-intuitive, it is widely used in Bangladesh to describe monsoon season field inundation depth and micro-elevational differences in rice growing areas. The development of crop management practices that can increase yield and profitability for *aman* rice grown on highlands and medium-highlands (which when combined represent 66% of coastal land area), is therefore crucial. Yet despite policies aimed at boosting *aman* rice productivity (e.g. USD 306 million has been requested from international donors to support agricultural development in coastal Bangladesh (MoA and FAO, 2013)), relatively little applied research has been conducted on farmers’ fields to identify improved management practices suitable to these landscape positions.

Increased fertilizer application – particularly nitrogen (N) – is perhaps the most widely applied strategy for enhancing yield, but more than half of applied N is typically lost to the environment. Improving N use efficiency is particularly challenging in flooded rice where water depth can be deep, but may also fluctuate during the course of the growing season (Ladha et al., 2005). Low N recovery efficiency by aboveground biomass is has been documented in rice (Chien et al., 2009). This lower efficiency is caused by denitrification and volatilization, and in some environments may also result from leaching, seepage, and/or runoff, each of which are affected by farmers’ water and crop management practices (Choudhury and Kennedy, 2005).

Much of central coastal Bangladesh experiences monsoon season field inundation and relatively stagnant water in on medium-highlands (Krupnik et al., 2017). Limited information regarding N requirements is however available for rice grown under different landscape positions and flooding depths in these unique environments. Improved varieties are typically grown in highland fields. This contrasts with traditional varieties predominantly grown on medium-highlands. Numerous studies in more favorable environments have focused on N response for improved rice varieties developed by the Bangladesh Rice Research Institute (Kirttania et al., 2013; Masum et al., 2008; Chowdhury et al., 2016; Islam et al., 2014; Salahuddin et al., 2009), although less on-farm research on nutrient requirements for traditional varieties has been conducted, exempting select studies (e.g., Mamun et al., 2016).

Fertilizer N source can also influence N use efficiency in flooded rice systems (Ladha et al., 2005). In Bangladesh, prilled urea is the most popular N source, in part because it is supported by government subsidies. However, deep placement of urea in the form of urea super granules (USG) has been proposed to improve N efficiency by maintaining N in reduced soil layers, thereby limiting N transformations and losses (Gaihre et al., 2015; Huda et al., 2016). In this way, USG represents both a different source and placement method compared to prilled urea that is typically broadcast. These issues must considered when evaluating the relative performance of USG compared to prilled urea. Recent research in more favorable production environments in Bangladesh has shown that USG placed manually into the soil can increase N use efficiency compared to broadcast prilled ureas. Positive net economic benefits and 20% higher yields have also been observed (Miah et al., 2016; Huda et al., 2016). However, uncertainty remains about the economic efficiency of USG in different environments. Little is also known about the compatibility of USG with different types of varieties, for example traditional or improved high yielding cultivars. In much of Bangladesh, USG is also placed into the soil by hand after crop establishment to every-other alternate row of transplanted rice. In addition to requiring farmers to invest in all urea in the first two weeks of the cropping season, this requires an additional field operation for which labor and human energy are required. These considerations therefore must be weighed against potential yield increases. Considering that labor scarcity and cash liquidity are major constraints faced by *aman* rice farmers (USDA FAS, 2018), labor requirements rather than gains in yield or N use efficiency are to strongly influence farmers' decision to adopt USG-based N management.

Sustainable intensification (SI) refers to strategies for increasing food production on existing agricultural land while minimizing environmental impacts and maximizing the flow of ecosystem services (Pretty et al., 2011). Poorly managed N application can be associated with environmental externalities including greenhouse gas emissions. Nitrous oxide (N_2_O) produced during nitrification and denitrification processes is a particularly important greenhouse gas (GHG) associated with rice production. This gas is 265–298 times more atmospherically reactive than CO_2_. N_2_O also contributes to ozone-depletion (IPCC, 2006). Of an estimated 196.93 megatons CO_2_ equivalent of GHGs emitted during 2014, 13% came from rice fields (WRI, 2018; FAO, 2018). Application of N to fields subjected to extended periods of drying and re-flooding can also increase N_2_O emissions (Gaihre et al., 2015). Exempting work by Gaihre et al. (2017; 2015), *in-situ* measurements of N_2_O emissions are rare in Bangladesh. Gaihre et al. (2018) also found no effect of USG compared to prilled urea on N_2_O emissions in well-managed highland *aman* rice fields with careful water control. Conversely, to our knowledge, no measurements of emissions from alternative N fertilizer sources have been made in Bangladesh’s central coastal region.

Energy use efficiency is another critical indicator for SI. Fossil fuel use for tillage and fertilizer production both result in GHG emissions (Soni et al., 2013). In Bangladesh, energy used by agriculture and forestry increased from 0.2% to 5.1% of total energy consumption between 1971 and 2009 (FAO, 2018). Energy inputs to agriculture increased 51% from 1990 to 2005, although cereal production increased only 35% (Khosruzzaman et al., 2010). This is indicative of a decline in efficiency. Modifications in N management can also increase energy efficiency either by reducing energy inputs and/or increasing yields (Yadav et al., 2017), although this subject also remains understudied in coastal Bangladesh’s rice agroecosystems.

We addressed these concerns by conducting a multi-criteria evaluation of the potential costs and benefits of N management in farmers’ fields in the central coast of Bangladesh. Our objectives were to evaluate the effects of N rate and source on the agronomic, economic, and environmental performance of *aman* rice grown on both highland and medium-highland landscape positions. We sought to (1) quantify grain yield response to prilled and USG in different landscape positions, (2) measure economic performance through farmer-participatory trials, and (3) assess environmental performance by estimating GHG emissions and energy use efficiency.

## 2 Materials and methods

### 2.1 Study area and experimental design

**Fig. 1:**
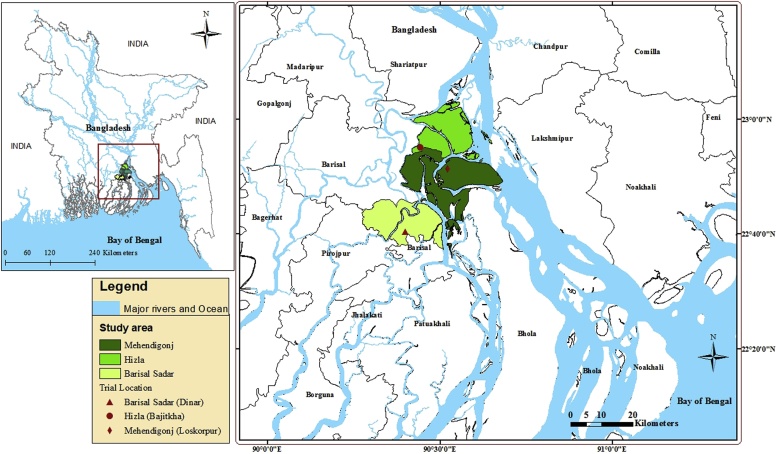
Map of study area located in the Barisal district of coastal Bangladesh. The three sub-districts: Barisal Sadar, Hizla, and Mehendigonj are low elevation (1–3 ms l). The latter sub-districts are coastal islands.

Multi-locational on-farm trials were conducted during the 2013 *aman* season in three sub-districts of Barisal district on the central coastal Bangladesh. Barisal Sadar, Hizla and Mehendigonj sub-districts are located in the Ganges Tidal Floodplain (with silty clay soils with low N and organic matter concentrations (FRG, 2012)). Transplanted *aman* (T. *aman*) rice is rainfed and benefits from tidal fresh water ebb and flow in this region. Farmers in each location were selected because of their willingness to participate in researcher-designed but farmer-managed experiments (Fig. 1). Two landscape positions were selected for study, including highlands and medium-highlands (mean monsoon *aman* season field inundations depths <30 cm or 30–90 cm, respectively) (Brammer, 2012). Depending on the frequency of precipitation and tidal water movement, fields in both landscapes may experience considerable fluctuation in water depth, and even periods without standing water.

Fifty-one farmers volunteered to participate in this study, 30 with fields on highlands and 21 in medium-highlands. Farmers’ fields were dispersed over each landscape, with each considered a replicate (*n* = 10 and 7 for highlands and medium-highlands, respectively, within each sub-district). Farmers opted to grow the most dominant rice variety that is appropriate for each landscape position. These included BRRI *dhan* 39 for highland fields and *Bhushiara* for medium-highland fields. BRRI *dhan* 39 is a semi-dwarf high yielding cultivar released in 1999 and is considered suitable for highlands. This variety may suffer mortality if subjected to flooding for prolonged periods and therefore cannot be reliably grown in medium-highland or lower lanscape positions. As such, ‘improved’ high yielding varieties like BRRI *dhan* 39 have not been widely adopted by farmers on medium-highlands in coastal environments. Farmers with fields on medium-highlands therefore selected *Bhushiara*, a traditional and high-tillering variety commonly grown to produce puffed rice, for these experiments.

Farmer-repetitions were nested within sub-districts. Although they were dispersed, no single field was more than 1 km from its furthest neighbor. N source (prilled urea and urea super granules) and N rate were imposed in ach farmers’ field in a split-plot format. BRRI *dhan* 39 and *Bhushiara* typically have different N rate recommendations specific to each landscape position. The recommended N rate for high yielding transplanted *aman* rice for the study area is 75 kg N ha^−1^ (FRG, 2012), while the recommended N rates for traditional varieties are 0–15, 16–30, 31–45, and 46–60 kg N ha^−1^ for optimum, medium, low, and very low soil fertility levels, respectively (FRG, 2012). Our trials conversely considered potential interactions between N source and rate. In highland trials, N was therefore applied to BRRI *dhan* 39 at 0, 25, 50, and 75 kg N ha^−1^, while in medium-highland trials it was applied to *Bhushiara* at 0, 28, 42 and 56 kg N ha^−1^. N source plots were 10.75 m × 8.75 m in highlands and 12.75 m × 8.75 m in medium-highlands, with individual rate sub-plots measuring 5 m × 4 m in highlands and 6 m × 4 m in medium-highlands.

Rice seedlings were raised following standard practices. Land preparation in each field included conventional rotary tillage followed by laddering with three passes of a two-wheel tractor. For highland and medium-highland trials, respectively, 20–25 and 35–40 days old seedlings were transplanted at a spacing of 20 cm × 20 cm and 25 cm × 30 cm. Transplanting older and taller seedlings of traditional varieties is a common practice to improve seedling establishment in flood-prone areas under which younger seedlings are likely to experience submergence and mortality. The following basal fertilizers were also applied: 20 kg P ha^−1^, 25 kg K ha^−1^, 5.33 kg S ha^−1^, 5.7 kg Ca ha^−1^ and 0.46 kg Zn ha^−1^ for highland, and 6 kg P ha^−1^, 4 kg K ha^−1^, 5.33 kg S ha^−1^, 5.7 kg Ca ha^−1^ and 0.46 kg Zn ha^−1^ on medium-highlands as Triple Superphosphate (TSP), Muriate of Potash (MoP), CaSO_4_, and ZnSO_4_, respectively. In both medium-highlands and highland trials, 100% USG was applied at 10–15 days after transplanting (DAT). N rates of 25, 50, and 75 kg ha^−1^ were obtained by manually placing urea balls weighing 0.9, 1.8, and 2.7 g each at 4–8 cm depth in the center of four hills in alternating rows in highland trials (4 hills therefore received 1 super granule). In medium-highland trials this corresponded to 1.8, 2.7, and 2  ×  1.8 g USG balls for the N rate treatments of 28, 42, and 56 kg N ha^−1^, respectively. Prilled urea was applied in two equal splits at 10–12 DAT and at panicle initiation in medium-highland trials. On highlands, prilled urea was applied in three splits: 25% at 10–12 DAT, 37.5% at 25 DAT, and 37.5% at panicle initiation. Trials were regularly weeded and no pests were observed.

### 2.2 Data collection

**Fig. 2:**
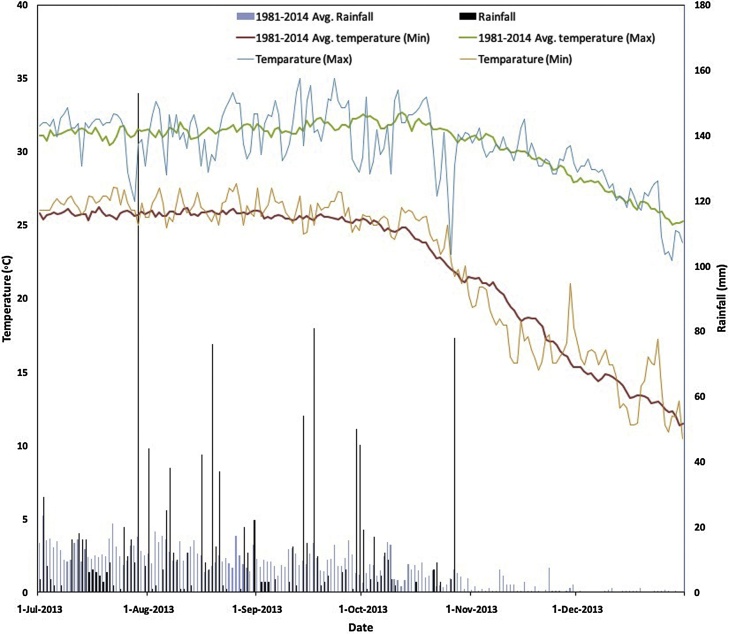
Temperature and rainfall in Barisal district of Bangladesh during the study period (2013 *aman* season) and the 1981–2014 medium-term average.

To determine baseline soil characteristics at each site, three composite samples from each plot were collected at 0–15 cm depth at the beginning of the experiment and analyzed following standard procedures (Table 1). N, available P, exchangeable K, and pH were all measured as described by SRDI (2014). Exchangeable K was determined by atomic absorption spectroscopy after 1 M NH4OAc extraction at pH 7. Dates of crop management and phenology were recorded in each field. Grain yields were collected from a 5 m^2^ area within each plot and were moisture corrected to 14%. Daily weather data during the growing season were collected from an automatic weather station (Fig. 2).

**Table 1:** Summary of monsoon season inundation depth and baseline soil characteristics at the 0–15 cm depth for each Sub-District in this study^1^.

Landscape position	Description	Sub-District	pH	OM (%)	Exchangeable K (mg kg^−1^)	Total N (%)	P (μg g^−1^)	S (μg g^−1^)	Zn (μg g^−1^)
Highlands	Inundated up to 0-30 cm depth on average during the monsoon	Barisal Sadar	7.70	0.55	46.8	0.03	9.83	17.46	0.34
		Hizla	7.63	0.50	58.5	0.03	10.33	22.06	0.24
		Mehendigonj	7.06	0.95	97.5	0.06	9.58	32.50	0.34
Medium-highlands	Inundated between 30-90 cm on average during the monsoon	Barisal Sadar	7.80	0.88	42.9	0.05	10.38	14.76	0.24
		Hizla	7.69	0.98	70.2	0.06	8.11	28.86	0.20
		Mehendigonj	7.65	1.05	66.3	0.06	11.09	25.11	0.24

1 N, available P, exchangeable K, and pH were all measured as described by SRDI (2014). Exchangeable K was determined by atomic absorption spectroscopy after 1 M NH4OAc extraction at pH 7. Soils were not subject to salinity analysis as research locations are known to be outside of zones with coastal salinity problems (cf. Krupnik et al., 2017).

### 2.3 Economic and environmental indicators

Each farmer was questioned following crop management operations to determine the costs of land preparation and agronomic inputs, as well as labor costs. Labor requirements for field operations were observed for all plots and timed by researchers with stopwatches. Economic efficiency (ECE) was estimated as the ratio of total returns to total variable costs. Total returns were calculated as the product of grain yield and reported rice price. BRRI *dhan* 39 and *Bhushiara* prices were estimated at USD 211.1 and 241.25 t^−1^, respectively. Total variable costs of production were calculated including all land preparation, inputs and labor costs (Supplementary Tables 1–6).

The energy efficiency of inputs and outputs was estimated using common energy conversion coefficients for each farmer-replicate (Table 2). Although these coefficients may vary due to differences in assumptions and spatial and temporal system boundaries (Hülsbergen et al., 2001), this approach is widely employed in the literature to evaluate relative treatment differences. Energy parameters were calculated on a per hectare basis from sowing to harvesting and included transport of the harvested product to farmers’ houses by laborers. Energy use for land preparation was calculated based on fuel consumption estimates. Economic and embedded energy costs for land preparation machinery were however considered to be foregone and were not included in our economic analyses. Required energy for mechanically converting prilled urea to urea super granules was calculated based measurements of fuel consumed (1.7 l diesel h^−1^) to produce approximately 370  kg USG as reported by International Fertilizer Development Center staff in Bangladesh (Mahmud et al., 2017).

**Table 2:** Energy conversion factors used in this study.

		MJ unit^−1^	Source
Material inputs	Diesel (L)	47.70	Pimentel and Pimentel (2008) and Quilty et al. (2014)
	Nitrogen (N kg)	66.14	Rahman and Hasan (2014)
	Phosphorus (P_2_O_5_ kg)	12.44	Rahman and Hasan (2014)
	Potassium (K_2_O kg)	11.15	Rahman and Hasan (2014)
	Seed (kg)	15.50	Pimentel and Pimentel (2008)
	2-wheel tractor operator (h)	0.98	Ainsworth et al. (2011) and Quilty et al. (2014)

Human labor	Transplanting (h)	0.79	Ainsworth et al. (2011) and Quilty et al. (2014)
	Broadcast fertilizer application in wet soils (h)	0.98	Ainsworth et al. (2011) and Quilty et al. (2014)
	Urea super granule application (h)^a^	0.98	Ainsworth et al. (2011) and Quilty et al. (2014)
	Manal weeding (h)	0.96	Ainsworth et al. (2011) and Quilty et al. (2014)
	Manual rice harvesting (h)	0.89	Ainsworth et al. (2011) and Quilty et al. (2014)

Outputs	Rice grain yield (kg)	15.20	Pimentel and Pimentel (2008)

a Application of urea super granules requires similar labor and physical movement as transplanting. Farmers bend over and place USG at 5–10 cm depth into the soil-among rice hills. We therefore assumed the same conversion factor as transplanting because of a lack of direct measurement and hence coefficient of energy use for manual USG application. Application however may take less time than transplanting as granules are placed in alternating rows. This descrepency was accounted for in our data collection as alllabor operations were timed.

Energy efficiency parameters including agronomic energy input (AEI, Eq. (1)), grain yield energy (GYE, Eq. (2)), and net energy yield (NEY, Eq. (3)) were calculated. The proportion of AEI directly related to agronomic inputs and human labor was also determined,

(1) $$AEI=\sum ({AEI}_{a}+{AEI}_{mi})$$

(1a) $${\text{A}\text{E}\text{I}}_{\text{a}}\;=\frac{I_{a}\times {\;EF}_{a}}{A}$$

(1b) $${AEI}_{mi}=T\;x\;{EF}_{mi}$$

where *I*_*a*_ is the mass of an agronomic input “*a*” applied to a field with an area ‘*A*’ (ha), *EF_a_* is embedded energy for “*a*” (MJ kg^−1^ or MJ L^−1^), *T* is time (Person-hours) and *EF_mi_* is the energy factor of human labor (MJh^−1^) (Table 2). GYE was next computed as

(2) $$GYE=GY\;x\;{EF}_{gy}$$

where *GY* indicates grain yield (t ha^−1^) and *EF_gy_* is the energy factor of rice grain on a weight equivalent basis (Table 2). Finally, NYE was calculated as follows:

(3) $$NEY=\;{GYE}_{i}-\;{AEI}_{ti}$$

where *GYE_i_* is the grain yield energy of rice crop “*i*” calculated as in Eq. (2) and *AEI_ti_* is total energy input for the rice crop.

The relative impact of N application on partial GHG emissions was calculated as GHG efficiency (GHGE), defined as kg CO_2_ equivalent (CO_2_eq.) per kg of rice produced. Only partial GHG emissions and their corresponding CO_2_e were considered in this research as our study focused on field-based agronomic practices and yield performance (Table 3). Hence, we were unable to consider methane (CH_4_) emissions resulting from soil-water mediated biological processes, though we did account for CO_2_, N_2_O and CH_4_ emissions associated with the production and use of chemical fertilizer and fuel. For N_2_O, we considered both direct and indirect emissions associated with estimated N losses by following established methodology (cf. IPCC, 2006). Table 3 provides the coefficients used for converting material inputs to CO_2_e based on their estimated global warming potential (GWP).

**Table 3:** Coefficients for greenhouse gas (GHG) emissions from agricultural inputs used in this study by following the IPCC (2006) and Lal (2004).

Emission source		GHG	Emission coefficients	Unit
Production, transportation and storage of fertilizers^1^	Nitrogen (N)	CO_2_	4.77	Kg CO_2_e kg^−1^ N
	Phosphorus (P_2_O_5_)	CO_2_	0.73	Kg CO_2_e kg^−1^ P_2_O_5_
	Potassium (K_2_O)	CO_2_	0.55	Kg CO_2_e kg^−1^ K_2_O
Diesel^2^		CO_2_	0.0741	Kg CO_2_e MJ^−1^
		CH_4_	0.000087	Kg CO_2_e MJ^−1^
		N_2_O	0.00887	Kg CO_2_e MJ^−1^
Direct N_2_O from N inputs (synthetic fertilizers) from flooded rice fields^2^		N_2_O	6.33	Kg CO_2_e kg^−1^ N
Indirect losses of N fertilizer from soils^2^	Leaching or runoff	N_2_O	1.096	Kg CO_2_e kg^−1^ N
	Volatilization and re-deposition	N_2_O	0.487	Kg CO_2_e kg^−1^ N

1 Values are obtained and converted from Lal (2004).

2 Values are obtained and converted from IPCC (2006).

Four sustainability indicators for energy productivity (EP), agronomic N use efficiency (ANUE), GHG efficiency (GHGE), and economic efficiency (ECNE) were calculated as follows:

(4) $$EP=\frac{Grain\;yield\;(kg\;{ha}^{-1})}{Agronomic\;energy\;input\;(MJ\;{ha}^{-1})}$$

(5) $$ANUE=\frac{Yield\;in\;plots\;with\;N\;\left(kg\;{ha}^{-1}\right)-\;yield\;in\;plots\;without\;N\;\left(kg\;{ha}^{-1}\right)}{N\;rate\;(kg\;N\;{ha}^{-1})}$$

(6) $$GHGE=\frac{GHG\;emissions\;\left(kg\;CO_{2}e\;ha^{-1}\right)}{Grain\;yield\;(kg\;ha^{-1})}$$

(7) $$E\text{C}NE=\frac{Total\;return\;({USD\;ha}^{-1})}{Total\;cost\;of\;production\;({USD\;ha}^{-1})}$$

### 2.4 Data analysis

Statistical analysis was performed using JMP (Ver. 14.1). Where data violated normality assumptions acording to the Shapiro-Wilk test, transformations were conducted to meet assumptions. Data were for each response variable were analyzed by employing a hierarchical mixed model with restricted maximum likelihood to estimate variance components. The model was set considering replication nested within sub-district, both treated random effect. N source and N rate were considered as fixed effects. Where *F* tests indicated significance, means were separated at alpha = 0.05 according to the Student’s-*t* test (for N source) or Tukey’s HSD for all other factors and their interactions.

## 3 Results

Both N source and rate had significant effects (*P <* 0.05 to 0.001) on all response variables in highland landscape positions (Table 4). The N source × rate interaction was also highly significant (*P* < 0.001) for all response variables. In medium-highland trials, we observed significant effects of rate (*P* < 0.001) on all variables, but effect of N source was consistently insignificant (Table 5). In highland trials where BRRI *dhan* 39 was grown, grain yield increased significantly with increasing N rate, regardless of N source (Table 4). In terms of N source, USG consistently produced 4.2–5.8% higher yields than prilled urea at all N rates. Considering the interactive effect observed, we found that yield (4.23 t ha^−1^) at 50 kg N ha^−1^ as USG gave similar yield (4.28 t ha^−1^) at 75 kg N ha^−1^ with prilled urea. In medium-highland trials where the traditional *Bhushiara* variety was grown, the highest grain yield was produced with 28 N kg ha^−1^ (Table 5). Increasing N rates beyond 28 kg N ha^−1^ negatively affected grain yield.

**Table 4:** Fixed effect of nitrogen rate and source on yield, agronomic N use efficiency (ANUE), economic efficiency (ECNE), net energy yield (NEY), energy productivity (EP), and greenhouse gas efficiency (GHGE) for experiments conducted on highland landscape positions.

		Yield	ANUE	ECNE	NEY	EP	GHGE
Effect	Treatment	(t ha^−1^)^a^	(kg grain kg N^–1^)^a^	(USD return USD cost^−1^)^a^	(GJ ha^−1^)^a^	(kg grain MJ^–1^)^a^	(kg CO_2_ eq. kg grain^−1^)^a^
N Source (S)	Prilled	3.64 b	19.98 b	1.57 b	50.47 b	0.80 a	0.153 a
	USG	3.77 a	25.69 a	1.58 a	52.42 a	0.82 b	0.147 b

N Rate (R)	0	2.87 d		1.35 c	41.13 d	1.10 a	0.04 d
(kg N ha^−1^)	25	3.48 c	23.91 a	1.50 b	48.65 c	0.83 b	0.13 c
	50	4.12 b	24.74 a	1.72 a	56.85 b	0.72 c	0.18 b
	75	4.37 a	19.86 b	1.74 a	59.16 a	0.60 d	0.25 a

S × R	Prilled, 0	2.90 f		1.36 d	41.49 f	1.11 a	0.04 g
	Prilled, 25	3.39 e	19.60 bc	1.48 c	47.39 e	0.81 c	0.13 e
	Prilled, 50	4.00 c	21.98 bc	1.69 b	55.14 c	0.70 e	0.19 c
	Prilled, 75	4.28 b	18.37 c	1.74 a	57.86 b	0.59 f	0.25 a
	USG, 0	2.85 f		1.34 d	40.76 f	1.09 a	0.04 g
	USG, 25	3.56 d	28.23 a	1.51 c	49.91 d	0.85 b	0.12 f
	USG, 50	4.23 b	27.50 a	1.74 a	58.57 ab	0.74 d	0.18 d
	USG, 75	4.46 a	21.35 b	1.74 a	60.46 a	0.61 f	0.24 b

*F*-values	Source	79.17***	66.89***	4.79*	76.46***	19.68***	90.01***
	Rate	498.44***	9.71***	223.5***	326***	499.4***	3386.47***
	S × R	20.50***	10.95***	6.31***	19.96***	12.47***	21.84***

*, **, and *** indicate significance at 0.05, 0.01, and 0.001 probability level.

a Letters in columns not separated by blank rows indicate differences at alpha = 0.05 according to the Student’s t (for N source) or Tukey’s HSD for all other factors and their interactions. Least Square Means separation indicated that random effects of location for Mehendigonj and Hizla were different than Barisal Sadar only for Yield, ANUE, ECNE, NEY, EP and GHGE.

**Table 5:** Fixed effect of nitrogen rate and source on yield, agronomic N use efficiency (ANUE), economic efficiency (ECNE), net energy yield (NEY), energy productivity (EP), and greenhouse gas efficiency (GHGE) for experiments conducted on medium-highland landscape positions.

		Yield	ANUE	ECNE	NEY	EP	GHGE
		(t ha^−1^)^a^	(kg grain kg N^–1^)^a^	(USD return USD cost^−1^)^a^	(GJ ha^−1^)^a^	(kg grain MJ^–1^)^a^	(kg CO_2_ eq. kg grain^−1^)^a^
Source (S)	Prilled	3.34	3.40	1.82	46.71	0.94	0.15
	USG	3.33	2.43	1.80	46.51	0.93	0.15

Rate (R)	0	3.31 b		1.94 a	48.16 b	1.55 a	0.03 d
(kg N ha^−1^)	28	3.70 a	13.83 a	1.98 a	52.30 a	0.95 b	0.12 c
	42	3.30 b	−0.27 b	1.77 b	45.38 b	0.70 c	0.19 b
	56	3.04 c	−4.80 c	1.55 c	40.60 c	0.54 d	0.27 a

S × R	Prilled, 0	3.29		1.94	47.94	1.55	0.03
	Prilled, 28	3.67	13.29	1.99	51.87	0.95	0.12
	Prilled, 42	3.33	0.96	1.76	45.97	0.71	0.19
	Prilled, 56	3.07	−4.05	1.59	41.10	0.55	0.27
	USG, 0	3.32		1.94	48.38	1.56	0.03
	USG, 28	3.73	14.36	1.97	52.73	0.96	0.12
	USG, 42	3.26	−1.50	1.78	44.80	0.68	0.19
	USG, 56	3.01	−5.56	1.51	40.15	0.53	0.27

*F*-values	Source	0.05 ns	0.27 ns	0.61 ns	0.06 ns	0.16 ns	0.09 ns
	Rate	18.98***	57.13***	34.24***	27.1***	398.57***	660.19***
	S × R	0.97 ns	1.02 ns	1.13 ns	1.00 ns	0.97 ns	0.23 ns

*** indicate significance at 0.001 probability.

a Letters in columns not separated by blank rows indicate differences at alpha = 0.05 according to the Student’s t (for N source) or Tukey’s HSD for all other factors and their interactions. Least Square Means separation indicated that random effects of location for Mehendigonj and Hizla were different than Barisal Sadar for Yield, ANUE, ECNE, and NEY.

In highland trials, higher N rates significantly decreased ANUE (e.g. an ANUE 19.86 kg grain ha^–1^ was obtained from at 75 kg N ha^–1^ vs. 24.74 kg grain ha^–1^ from 50 kg N ha^−1^) (Table 4). When comparing N source by rate, USG had higher ANUE than the prilled urea at all directly comparable rates. Conversely, in medium-highland trials, the lowest N rate (28 kg N ha^−1^) resulted in the highest ANUE, with values decreasing significantly as N rate increased due to reduced grain yield that appeared to be related to lodging observed at higher N rates (Table 5).

Economic efficiency improved in highland trials with increasing N rate up to 50 kg N ha^−1^ (Table 4). However, there were no differences between 50 and 75 kg N ha^−1^ which both had ECNE values of 1.74. For medium-highland trials, the highest ECNE was observed at 0 and 28 kg N ha^−1^, with significant decline beyond 28 kg N ha^−1^ (Table 5). Differences in variable costs largely depended on labor, which contributed 53–58% and 57–65% of total costs in highland and medium-highland trials, respectively (Supplementary Tables S1–S6). In both studies, USG deep placement required 2–3 more labor days ha^–1^than broadcasting PU.

In highland trials, NEY increased with N rate, and USG was found to consistently improve NEY compared to prilled urea (Table 4). For farmers with medium-highland fields, lower N rates (0 or 28 kg N ha^−1^) resulted in similar NEY values as higher N rates (Supplementary Table S5). Application of N fertilizer conversely decreased EP (the ratio of grain yield to AEI) in both landscape positions (Table 4, Table 5). In fields located on highlands with BRRI *dhan* 39, USG was found to consistently improve EP compared to prilled urea except at 75 kg N ha^-1^ (Table 5). No differences in were however found on medium-highlands with *Bhushiara*, regardless of rate.

In both medium-highland and highland trials, GHGE significantly increased with increasing N rate. The lowest GHGE values were observed without N addition, whereas GHGE typically increased by six times at the highest N rate in highland trials (Table 4). GHGE conversely increased by nine times at the highest N rate in medium-highland trials (Table 5). In highland trials, USG was found to consistently improve yield-scaled greenhouse gas emissions compared to prilled urea.

The contribution of N fertilizer to total GHG emissions was 73, 84, and 89% for 25, 50, and 75 kg N ha^−1^, respectively, in highland landscape positions. In medium-highland trials, N fertilization contributed 81, 87, and 90% to emissions for 28, 42, and 56 kg N ha^−1^, respectively. Similarly, in both experiments, AEI was also heavily influenced by N rate. On highlands with BRRI *dhan* 39, N fertilizer contributed to 36, 53, and 63% of total AEI at 25, 50, and 75 kg N ha^−1^, whereas observed percentages were 44, 54, and 61% of total AEI at 28, 42, and 56 kg N ha^−1^, in medium-highland with Bhushiara, respectively (Supplementary Tables S5 and S6).

## 4 Discussion

### 4.1 Grain yield and economic performance

Considering the necessary N rate to increase yields and profit for *Bhushiara* grown on medium-highlands in coastal Bangladesh, the results of our preliminary study indicate that current extension recommendations provided by the Bangladesh Agricultural Research Council may require rethinking. We found that yields were highest at 28 kg N ha^−1^, while profits remained unchanged at 0 and 28 kg N ha^−1^. There was also a tendency for yield and profit to decline at 42 or 56 kg N ha^−1^. In contrast, current recommendations for traditional rice varieties in the study area are 45 kg N ha^−1^ for ‘low fertility’ soils with a target yield of 3 t ha^−1^ (FRG, 2012). While caution is required because our results are based on limited observations with a single yet widely popular traditional variety, our multi-criteria evaluation of N application, which goes beyond typical recommendation frameworks to consider the environmental costs of applying excess fertilizer, suggests it may be beneficial to conduct further research to refine N rate recommendations for traditional rice varieties on medium-highland fields.

One reason for this discrepancy may be that in research station environments commonly used to develop N rate recommendations may differ in nutrient concentrations when compared tounbalanced application in farmers’ fields. Research station trials also tend to be meticulously managed by researchers, and as such may differ from trials managed by farmers on their own fields (de Roo et al., 2017). We also observed that *Bhushiara* tended to lodge at higher N rates. This appears to be a consequence of this variety’s taller stature, which while being well-suited to the fluctuating water depths found in medium-highlands subject to tidal ebb and flow, appears to be less suitable to support higher grain yield without lodging. Although rice production in medium-highland fields can be considered as ‘low-input’ because farmers typically apply little to no N fertilizer to traditional varieties, progressive soil fertility depletion suggests that some level of supplemental fertilization and improved organic matter management is likely to be required in the future (Shelley et al., 2016).

Due to the lack of research on traditional varieties under on-farm conditions across multiple locations, our preliminary results represent an important initial step in developing data-driven management practices. No yield responses were observed in medium-highland environments, as farmers applied N either as prilled urea or USG. Hence, more research is likely to be required in efforts to improve N fertilizer management for the *aman* season in coastal Bangladesh. One approach could be to estimate indigenous soil N supply to guide the development of site-specific nutrient management practices for individual farmers’ fields considering their cropping and soil management history, as well as attainable yields (Ladha et al., 2016). Consideration of other macro- and micro-nutrients are also areas for additional research. Trade-offs associated with the cost to extension services of deploying field-specific recommendations to farmers in population-dense countries like Bangladesh however should be carefully weighed. Current recommendations in the study area are based on yield goals and soil fertility categories derived from soil tests (FRG, 2012). Indigenous soil N supply can however fluctuate greatly in lowland rice agroecosystems (Cassman et al., 2002). Given current interest among policy makers to increase N use efficiency, which could offset large public subsidy requirements for fertilizers, increased knowledge of indigenous soil N supply could help guide farmers’ decisions, particularly from a profitability standpoint. For example, our results indicate that N rates in medium-highland fields may be reduced to avoid inefficiencies while still maintaining profitability. Additional work should nonetheless be conducted to confirm these observations.

The recommended N rate for improved varieties like BRRI *dhan* 39 grown in highland landscape positions is 61–80 kg N ha^−1^ for very low fertility soils with a yield target of 4 t ha^−1^ (FRG, 2012). Our results generally support this recommendation, as 75 kg N ha^−1^ tended to maximize yield. Previous research in Mymensingh in north central Bangladesh has documented that transplanted *aman* rice showed improving yield with increasing N rate up to approximately 55–60 kg N ha^−1^ in the form of USG, or 75–150 kg N ha^−1^ as prilled urea. This response was however dependendt on variety and other inter-cultural practices (Kirttania et al., 2013; Masum et al., 2008; Chowdhury et al., 2016; Islam et al., 2014; Salahuddin et al., 2009).

Not all studies, however, have considered the risk of reduced profitability from increased labor and costs for USG application. In our study, manual placement increased labor requirements by three to four person-days ha^–1^ across sub-districts and landscape positions relative to broadcasting prilled urea. While these increases are relatively small in comparison to other operations such as transplanting, they represent a departure from conventional intercultural management practices in Bangladesh’s increasingly scarce and costly rural labor environment (Mottaleb et al., 2016). Rural laborers who are often hired to apply fertilizer by broadcasting may also find manual USG placement onerous. Options to mechanize USG placement with small-scale equipment have therefore been undertaken to address this concern (Chatterjee et al., 2018), though they have not yet been made commercially available and as such as are not widely adopted. Larger-scale equipment is also unlikely to be appropriate for the small-field sizes and limited resource endowments of farmers in Bangladesh (Krupnik et al., 2013; Mottaleb et al., 2016). Resource-poor farmers with cash liquidity constraints at the beginning of the season may also experience difficulty applying urea super granules, which require up-front investment in all N fertilizer at the beginning of the season, rather than purchasing and metering N in splits over time.

In the southwest of coastal Bangladesh, which is subject to less tidal water influence, yield increases in response to N have been observed up to approximately 95 kg N ha^−1^ (Zaman et al., 2007). Rice yield increases following N application in more favorable highland environments and non-coastal regions have been found to be similar to the northern part of Bangladesh (Sidhue et al., 2004). In our study, yields generally did not reach a plateau at 75 kg N ha^−1^ on highlands with BRRI *dhan* 39. This suggests higher N rates should be included in future research. Although the highest N rate generally produced maximum yield, from a profitability standpoint, economic efficiency plateaued at 50 to 75 kg N ha^−1^ on highlands with BRRI *dhan* 39. This trend was found regardless of N source. Based on only agronomic and economic criteria, this research therefore provisionally supports the recommendation of 75 kg N ha^−1^ as prilled or USG to achieve maximum yield without sacrificing profitability on highlands with BRRI *dhan* 39. However, our data also inficate that farmers may consider using 50 kg N ha^−1^ as USG to achieve similar grain yield as with 75 kg N ha^−1^ applied as prilled urea. These results appear to be obtainable without affecting economic efficiency on highlands with BRRI *dhan* 39. In coastal Bangladesh, where both rice productivity and economic profitability are of major concern – as both affect household food security – identifying a balance between agronomic, economic, and environmental outcomes is an important first step towards meeting SI goals.

We found no significant effect of USG on yields in medium-highland fields, but a significant effect of N source and rate as well as their interaction in fields managed by farmers in highland landscape positions. In these locations, USG consistently produced 0.17–0.23 t ha^−1^ higher yields than prilled urea at N rates of 50 and 75 kg N ha^−1^. These support other observations where deep placement of urea has been found to increase yield compared to prilled urea in favorable environments and with improved varieties (Kirttania et al., 2013; Chowdhury et al., 2016; Islam et al., 2014; Masum et al., 2008). We were however not able to measure the potential effects of N deposition, tidal water flow, or seepage in our experiments. Further research over multiple seasons that considers these and additional components of overall N balances may be useful to fine-tune nutrient management recommendations and reduce risk of environmental externalities.

When evaluating profitability instead of yield, our preliminary results however indicate limited economic benefit from USG. Notably, even in cases where yield increases were observed, the additional labor requirements of USG application offset the economic benefits associated with higher yields at 75 kg N ha^−1^ in both highlands and medium-highlands. It should be acknowledged that the yield increase with USG in our study locations was smaller than previous studies showing that USG can increase yield at the same rate as broadcast prilled urea, while reducing N losses by around one-third (Kirttania et al., 2013; Miah et al., 2016). One possible explanation is that the overall magnitude of N response was relatively low in both highland and medium-highland trials, potentially masking any advantage of USG. Despite the potential for yield increases and reduced environmental N losses, farmers are not likely to adopt USG if labor costs are high and profits are limited. Miah et al. (2016) conversely found positive net economic benefits of USG based on an average yield increase of 21% across 41 farmer fields in the *aman* season. These contrasting results suggest further work is needed to understand if labor requirements and limited profitability prospects help explain the relatively low adoption of USG in Bangladesh, despite large extension and development support in recent decades.

Yields for trials in this study were higher than the average for the Barisal district (in which the three sub-districts were included) during the *aman* season (1.64 and 2.67 t ha^−1^ for *Bhushiara* and BRRI *dhan* 39, respectively) (BBS, 2016). Although current development efforts aim to increase the adoption of improved varieties in this region, our results may provide insights as to why farmers may prefer local varieties for risk-prone medium-highlands in coastal Bangladesh. It is estimated that there are more than 1000 cultivars of local varieties in Bangladesh. Many are popular due to their wide adaptability in stress-prone situations, superior quality, and higher market price (Shelley et al., 2016). Hossain et al. (2006) pointed out that minor differences in landscape elevation that affects the depth of floodwater in the *aman* season is a main constraint for adopting short-statured and high-yielding varieties. This observation may help to explain why these varieties occupy only around half of Bangladesh’s *aman* area, though predominantly on highlands.

### 4.2 Yield scaled emissions, net energy yield, and energy produtivity

In South Asia’s coastal zones, development efforts tend to focus on disaster preparedness and agricultural productivity interventions. The latter is often centered on cropping systems intensification, though potential environmental costs including energy consumption and GHG emissions should also considered. Cropping systems intensification tends to rely on increased levels of mechanization and the adoption of improved varieties (Mottaleb et al., 2016); the latter usually have higher N requirements. We observed that increasing N rates on both medium-highlands and highland farmers’ fields were associated with large reductions in ANUE and EP alongsisde increasing greenhouse gas emissions. Although the N rates applied were quite low, the environmental risks associated with N fertilizer inputs for *aman* rice in this coastal environment should not be overlooked. At the same time, the contrasting responses observed for N addition on medium-highlands and highlands indicate that distinct opportunities likely exist for addressing SI goals in coastal Bangladesh, depending on underlying physical geography. As detailed below, few conflicts between agronomic and environmental goals were observed in medium-highlands with *Bhushiara* where low levels of N inputs were required to maximize yield. Yet significant tradeoffs were found for farmers in highland landscape positions where higher levels of N inputs were necessary to increase yield.

Regardless of landscape position, the largest contributor to energy consumption in our preliminary study was N fertilizer inputs (data not shown). This agrees with previous reports for other rice cropping systems (Quilty et al., 2014; Khan and Hossain, 2007; Rahman et al., 2015). Mechanization in Bangladesh’s coastal zone is slowly but steadily increasing (Mottaleb et al., 2016), which results in increased fuel use for land preparation. We calculated energy efficiency using two different methods, NEY and EP, each accounting for energy outputs as grain yield. Trends for NEY were similar to observed yield responses, with NEY inversely related N rate in medium-highland trials, but positively related in highland trials (Table 4, Table 5). While results for NEY mirrored grain yield, increasing N rate decreased EP for both highland and medium-highland trials, as EP represented the efficiency of energy inputs as a ratio with grain yield as the numerator. Values for EP in this study are greater than previous estimates in Bangladesh (Khosruzzaman et al., 2010). This may be explained by the relatively low energy inputs into this system (e.g. AEI 2.6 – 7.3 GJ ha^−1^). The lack of irrigation and relatively low fertilizer rates resulted in relatively high efficiency, even though grain yields were lower than other production regions. Examples of energy inputs and grain yield have been estimated for rice production include cropping systems in Borneo (AEI 4.3 GJ ha^−1^, yield 2.02 t ha^−1^), Japan (AEI 34.40 GJ ha^−1^, yield 6.33 t ha^−1^), Philippines (AEI 7.64 GJ ha^−1^, yield 1.654 t ha^−1^), and the United States (AEI 49.53 GJ ha^−1^, yield 7.367 t ha^−1^), with differences largely owing to the level of mechanization and external inputs (Pimentel and Pimentel, 2008). Findings from this study are in line with low energy input systems reported for rainfed rice in Thailand where yield was 2.63 t ha^−1^ and direct energy consumption was 5.01 GJ ha^−1^ (Soni et al., 2013), as well as studies in farmers’ fields during the monsoon in the Philippines where yield was 3.85 t ha^−1^ and energy consumption was 12.6 GJ ha^−1^ (Quilty et al., 2014).

The agronomic efficiency of N fertilizer is a reflection of both the yield potential of the production system and inherent soil fertility levels (Ladha et al., 2005). It is generally agreed that ANUE can be enhanced by reducing N fertilizer inputs, though tradeoffs with yield will typically result (Chen et al., 2011; Cassman et al., 2002). Although preliminary, our results provide further support for this concept with experiments in both highland and medium-highland environments having higher ANUE at lower N rates, and USG performing better at higher rates than prilled urea in highland trials. Hossain et al. (2005) found no relationship between agronomic efficiency and indigenous soil N supply in conditions where yield was limited by other factors. As discussed above, these results suggest that addressing other agronomic factors including proper transplanting methods, seedling age, plant density, and timing of fertilizer application may be necessary to increase overall yield potential and ANUE.

Partial GHG emissions were estimated based on fuel consumed for land preparation, the production of USG, and estimated direct and indirect N losses. Although methane can be an important source of GHG emissions in flooded rice fields, we were unable to measure emissions *in-situ* in our multi-location and dispersed replicate on-farm trials situated in relatively remote locations in coastal Bangladesh. We however did not include or vary organic matter inputs in our experimental design, and as this study focused primarily on fertilizer N inputs that are unlikely to affect methane emissions (IPCC, 2006). However, future research may benefit from quantification of methane emissions, either through modeling, *in-situ* measurement, use of coefficients or other methods. We were also unable to differentiate N source variation for estimating indirect N losses, although we did consider fuel consumption to convert prilled urea into USG. The latter measurement is usually not included in plot-based agronomic studies, the results of which still appear to be inconclusive.

N_2_O reductions of 40–50 % have been reported in Bangladesh when N applied as USG was 30% lower than prilled urea in the *aman* season on highlands with precise water control (Gaihre et al., 2015). In our study, BRRI *dhan* 39 grown on highland fields showed increased yields and ANUE response considering USG relative to prilled urea, in partial support of these observations. Gaihre et al., 2018 conversely reported similar seasonal N_2_O emissions from urea deep placement and prilled urea in *aman* season rice on lowlands. In our study, we observed no effect of N source in medium-highlands using *Bhushiara*, indicating the potential for similar losses. Using meta-analysis, Linquist et al. (2012) found that USG can contribute to N_2_O emissions, although further research on the effects of placement into reduced soil layers and floodwater depth are required.

Given the on-farm nature of this research, and the uncontrolled floodwater conditions experienced in our study environments, we were unable to directly quantify gaseous N losses from either form of urea fertilizer. We therefore applied a conservative estimate using the general conversion factors provided by the IPCC (2006). Our estimates of emissions however show that GHGE increased rapidly in response to increasing N rate in both highland and medium-highland trials, with increases in GHG emissions more than offsetting the significant yield increases observed on highlands. Compared to no N fertilizer, 28 kg N ha^−1^ increased GHGE by four times in medium-highland fields with *Bhushiara*. Application of 25 kg N ha^−1^ increased GHGE by three times in highland fields with BRRI *dhan* 39 (Table 4, Table 5), which has a larger sink capacity. The increase in GHG emissions in our study largely resulted from N application, including the production of N fertilizer, direct N_2_O emissions from the soil, and indirect N_2_O emissions resulting from other N loss pathways. Fertilizer inputs often make a larger contribution to GHG emissions compared to other inputs (Pokhrel and Soni, 2017), with GHG emissions also increasing at higher application rates. Without including methane emissions, Soni et al. (2013) reported 560.2 kg CO_2_eq. ha^−1^ GHG emissions from rainfed transplanted rice systems in Thailand with a grain yield of 2.63 t ha^−1^, resulting in a GHGE of 0.2 kg CO_2_ eq. per kg grain. This falls within the range of our results for 50 and 75 kg N ha^−1^ on highlands (Table 4). In contrast, grain yield was higher in our study, contributing to a lower GHGE for highland fields compared to transplanted rainfed rice in Thailand. Additional research in other locations, soil types, and over multiple seasons should nonetheless be implemented to confirm these observations.

Given that SI goals related to environmental sustainability and food security are often competing, multi-criteria analyses are needed to minimize tradeoffs and help inform public policy and agricultural investment decisions. When integrating agronomic and environmental variables in this study, the application of N fertilizer resulted in contrasting outcomes depending on rice production context (i.e. the combination of landscape position, rice variety, and study location). For medium-highland landscape positions and the traditional variety *Bhushiara*, application of N fertilizer beyond 28 kg N ha^−1^ did not increase yields. Rates above this always resulted in lower environmental performance in terms of decreasing N use and energy efficiency, in addition to higher GHG emissions. From an SI standpoint, the implications are that economic profitability and environmental quality can be maintained with lower than the recommended rate of N application for the local variety *Bhushiara* on medium-highlands. In contrast, clear conflicts between yield response and environmental performance variables were observed for highland trials. To meet productivity and economic objectives for BRRI *dhan* 39 grown on highlands, 75 kg N ha^−1^ tended to be necessary. However, given that increasing N rate can reduce ANUE and EP, our preliminary results indicate that USG 50 kg N ha^−1^ represented a better balance of maintaining economic profitability while avoiding environmental costs associated with greater application rates. Integrated assessments like those demonstrated by this paper can help identify opportunities for addressing food production and profitability concerns while keeping environmental impacts to a minimum.

## 5 Conclusions

Bangladesh’s coastal environments face numerous food production, economic development, and environmental sustainability challenges. To our knowledge, this study represents the first multi-criteria assessment of agronomic, economic, and environmental performance of N rate and source for *aman* rice in the coastal rice agroecosystems of south central Bangladesh. Despite concerns over decreasing soil fertility levels, our preliminary results suggest that lower N fertilizer rates may be required to increase yields while simultaneously achieving desirable economic efficiency and environmental outcomes for the traditional variety *Bhushiara* when grown on medium-highlands. This indicates a potential win-win situation, though confirmatory research over multiple seasons is advised. For the improved variety BRRI dhan 39 grown on highlands, a clear tradeoff between agronomic, economic, and environmental goals was observed. Our results indicate that 75 kg N ha^−1^ achieved high yields and profits. In contrast, ANUE and energy efficiency were inversely correlated with increasing N rate, while GHG emissions were positively related. This suggests that where agricultural policy planners wish to balance environmental with economic development concerns, lower N rates may be advisable. USG – which has been widely promoted by development programs and extension services in Bangladesh – did tend to increase yield in highland landscape positions, though the costs (particularly for labor) associated with USG generally resulted in similar profits as prilled urea. On highlands, farmers cultivating BRRI *dhan* 39 may use 50 kg N ha^−1^ as USG to produce similar yield as obtained with 75 kg N ha^−1^ of prilled urea without affecting economic efficiency, and also limiting environmental externalities. These results highlight that when assessing changes in crop management practices, multiple indicators can be beneficial to identify potential conflicts between environmental concerns, yield and profitability.
