# Supplementary material for: Agronomic, economic, and environmental performance of nitrogen rates and source in Bangladesh’s coastal rice agroecosystems
Source: Field Crops Res. 2019 Sep 1;241:107567. doi: 10.1016/j.fcr.2019.107567 (PMC6737986; doi:10.1016/j.fcr.2019.107567)
Supplement: Supplementary file 1 [file mmc1.docx]

**Supplementary Information**

**Table S1.** Mean economic inputs and outputs used to calculate profitability for trials conducted on highland landscape positions (1 USD = 82.9 Tk).

| Location | Example highland trial in Barisal Sadar | | | | | | | |
| --- | --- | --- | --- | --- | --- | --- | --- | --- |
| N (Kg ha^–1^) | 0 | | 25 | | 50 | | 75 | |
| Urea Type | Prilled | USG | Prilled | USG | Prilled | USG | Prilled | USG |
| Grain yield (t ha^–1^) | 3.00 | 2.94 | 3.51 | 3.65 | 4.09 | 4.30 | 4.32 | 4.47 |
| Harvest index | 0.42 | 0.41 | 0.43 | 0.44 | 0.46 | 0.46 | 0.46 | 0.47 |
| *Capital costs* |  | | | | | | | |
| Ploughing (USD ha^–1^) | 90.73 | | | | | | | |
| *Material inputs* |  | | | | | | | |
| Seedling (USD ha^–1^) | 30.16 | | | | | | | |
| Urea cost (USD ha^–1^) | 0.00 | 0.00 | 14.42 | 16.39 | 28.85 | 32.78 | 43.26 | 49.16 |
| TSP cost (USD ha^–1^) | 30.16 | | | | | | | |
| MoP cost (USD ha^–1^) | 9.05 | | | | | | | |
| Gypsum cost (USD ha^–1^) | 2.07 | | | | | | | |
| ZnSO_4_ cost (USD ha^–1^) | 2.47 | | | | | | | |
| *Labor (8-hour person-days ha^-1^)* |  | | | | | | | |
| Land preparation^a^ | 2.74 | 2.74 | 2.74 | 2.74 | 2.74 | 2.74 | 2.74 | 2.74 |
| Basal fertilizer application | 1.08 | 1.08 | 1.08 | 1.08 | 1.08 | 1.08 | 1.08 | 1.08 |
| Urea fertilizer application | 0.00 | 0.00 | 2.44 | 5.17 | 2.44 | 5.51 | 2.44 | 5.94 |
| Seedling uprooting, carrying and transplanting | 30.00 | 30.00 | 30.00 | 30.00 | 30.00 | 30.00 | 30.00 | 30.00 |
| Weeding | 15.38 | 15.38 | 18.00 | 17.17 | 16.35 | 15.63 | 16.54 | 17.27 |
| Harvesting and carrying for threshing | 22.85 | 22.79 | 22.91 | 22.72 | 22.72 | 22.79 | 22.72 | 22.79 |
| Labor wages (USD/psd) | 3.62 | | | | | | | |
| Total threshing cost (USD/ha  ) ^b^ | 25.34 | 24.78 | 29.63 | 30.79 | 34.56 | 36.31 | 36.5 | 37.78 |
| Total Variable cost (USD/ha) | 450.91 | 450.15 | 488.1 | 497.16 | 500.55 | 515.47 | 517.57 | 540.29 |
| Gross Margin (USD/ha) | 182.59 | 169.47 | 252.7 | 272.71 | 363.43 | 392.38 | 394.88 | 404.22 |

^a^ Required labor for two-wheel tractor operation and removal of rice stubble from the previous crop. Includes construction of bunds.

^b^  Crop harvest shared with thresher operators based on grain weight at 3 kg grain shared per 40 kg threshed.

**Table S2.** Mean economic inputs and outputs used to calculate profitability for trials conducted on highland landscape positions (1 USD = 82.9 Tk).

| Location | Example highland trial in Hizla | | | | | | | |
| --- | --- | --- | --- | --- | --- | --- | --- | --- |
| N (Kg ha^–1^) | 0 | | 25 | | 50 | | 75 | |
| Urea Type | Prilled | USG | Prilled | USG | Prilled | USG | Prilled | USG |
| Grain yield (t ha^–1^) | 2.93 | 2.87 | 3.43 | 3.57 | 4.08 | 4.33 | 4.41 | 4.57 |
| Harvest index | 0.40 | 0.41 | 0.42 | 0.44 | 0.46 | 0.46 | 0.46 | 0.47 |
| *Capital costs* |  | | | | | | | |
| Ploughing (USD ha^–1^) | 90.73 | | | | | | | |
| *Material inputs* |  | | | | | | | |
| Seedling (USD ha^–1^) | 30.16 | | | | | | | |
| Urea cost (USD ha^–1^) | 0.00 | 0.00 | 14.42 | 16.39 | 28.85 | 32.78 | 43.26 | 49.16 |
| TSP cost (USD ha^–1^) | 30.16 | | | | | | | |
| MoP cost (USD ha^–1^) | 9.05 | | | | | | | |
| Gypsum cost (USD ha^–1^) | 2.07 | | | | | | | |
| ZnSO4 cost (USD ha^–1^) | 2.47 | | | | | | | |
| *Labor (8-hour person-days ha^-1^)* |  | | | | | | | |
| Land preparation^a^ | 2.75 | 2.75 | 2.75 | 2.75 | 2.75 | 2.75 | 2.75 | 2.75 |
| Basal fertilizer application | 1.07 | 1.07 | 1.07 | 1.07 | 1.07 | 1.07 | 1.07 | 1.07 |
| Urea fertilizer application | 0.00 | 0.00 | 2.41 | 5.11 | 2.41 | 5.51 | 2.41 | 5.92 |
| Seedling uprooting, carrying and transplanting | 30.00 | 30.00 | 30.00 | 30.00 | 30.00 | 30.00 | 30.00 | 30.00 |
| Transplanting in missing hills | 0.26 | 0.26 | 0.26 | 0.26 | 0.26 | 0.26 | 0.26 | 0.26 |
| Weeding | 14.80 | 14.65 | 16.50 | 17.31 | 15.81 | 14.34 | 16.16 | 15.98 |
| Harvesting and carrying for threshing | 22.83 | 22.74 | 22.8 | 22.8 | 22.86 | 22.86 | 22.92 | 22.83 |
| Labor wages (USD/psd) | 3.62 | | | | | | | |
| Total threshing cost (USD/ha  ) ^b^ | 24.77 | 24.26 | 28.99 | 30.13 | 34.44 | 36.54 | 37.21 | 38.60 |
| Total Variable cost (USD/ha) | 449.27 | 447.68 | 481.80 | 498.32 | 500.13 | 512.03 | 518.07 | 539.04 |
| Gross Margin (USD/ha) | 169.87 | 158.81 | 242.86 | 254.84 | 360.95 | 401.37 | 412.16 | 426.02 |

^a^ Required labor for two-wheel tractor operation and removal of rice stubble from the previous crop. Includes construction of bunds.

^b^  Crop harvest shared with thresher operators based on grain weight at 3 kg grain shared per 40 kg threshed.

**Table S3.** Mean economic inputs and outputs used to calculate profitability for trials conducted on highland landscape positions (1 USD = 82.9 Tk).

| Location | Example highland trial in Mehendigonj | | | | | | | |
| --- | --- | --- | --- | --- | --- | --- | --- | --- |
| N (Kg ha^–1^) | 0 | | 25 | | 50 | | 75 | |
| Urea Type | Prilled | USG | Prilled | USG | Prilled | USG | Prilled | USG |
| Grain yield (t ha^–1^) | 2.77 | 2.76 | 3.23 | 3.47 | 3.83 | 4.06 | 4.11 | 4.32 |
| Harvest index | 0.39 | 0.39 | 0.42 | 0.43 | 0.45 | 0.46 | 0.46 | 0.46 |
| *Capital costs* |  | | | | | | | |
| Ploughing (USD ha^–1^) | 89.38 | | | | | | | |
| *Material inputs* |  | | | | | | | |
| Seedling (USD ha^–1^) | 30.16 | | | | | | | |
| Urea cost (USD ha^–1^) | 0.00 | 0.00 | 14.42 | 16.39 | 28.85 | 32.78 | 43.26 | 49.16 |
| TSP cost (USD ha^–1^) | 30.16 | | | | | | | |
| MoP cost (USD ha^–1^) | 9.05 | | | | | | | |
| Gypsum cost (USD ha^–1^) | 2.07 | | | | | | | |
| ZnSO_4_ cost (USD ha^–1^) | 2.47 | | | | | | | |
| *Labor (8-hour person-days ha^-1^)* |  | | | | | | | |
| Land preparation^a^ | 2.76 | 2.76 | 2.76 | 2.76 | 2.76 | 2.76 | 2.76 | 2.76 |
| Basal fertilizer application | 1.06 | 1.06 | 1.06 | 1.06 | 1.06 | 1.06 | 1.06 | 1.06 |
| Urea fertilizer application | 0.00 | 0.00 | 2.39 | 5.18 | 2.39 | 5.67 | 2.39 | 6.07 |
| Seedling uprooting, carrying and transplanting | 30.00 | 30.00 | 30.00 | 30.00 | 30.00 | 30.00 | 30.00 | 30.00 |
| Weeding | 14.69 | 15.38 | 17.09 | 17.31 | 15.52 | 14.69 | 16.78 | 16.76 |
| Harvesting and carrying for threshing | 22.80 | 23.05 | 22.96 | 22.86 | 22.92 | 22.89 | 22.96 | 22.92 |
| Labor wages (USD/psd) | 3.62 | | | | | | | |
| Total threshing cost (USD/ha  ) ^b^ | 23.41 | 23.27 | 27.31 | 29.27 | 32.35 | 34.29 | 34.72 | 36.50 |
| Total Variable cost (USD/ha) | 446.27 | 448.31 | 481.38 | 497.00 | 494.83 | 510.83 | 517.39 | 536.94 |
| Gross Margin (USD/ha) | 139.10 | 133.42 | 201.47 | 234.72 | 314.03 | 346.53 | 350.53 | 375.46 |

^a^ Required labor for two-wheel tractor operation and removal of rice stubble from the previous crop. Includes construction of bunds.

^b^  Crop harvest shared with thresher operators based on grain weight at 3 kg grain shared per 40 kg threshed.

**Table S4.** Mean economic inputs and outputs used to calculate profitability for trials conducted on medium-highland landscape positions in Barisal Sadar (1 USD = 82.9 Tk).

| Location | Example medium-highland trial in Barisal Sadar | | | | | | | |
| --- | --- | --- | --- | --- | --- | --- | --- | --- |
| N (Kg ha^–1^) | 0 | | 28 | | 42 | | 56 | |
| Urea Type | Prilled | USG | Prilled | USG | Prilled | USG | Prilled | USG |
| Grain yield (t ha^–1^) | 3.42 | 3.53 | 4.10 | 4.13 | 3.73 | 3.74 | 3.31 | 3.46 |
| Harvest index | 0.33 | 0.34 | 0.35 | 0.36 | 0.32 | 0.32 | 0.31 | 0.32 |
| *Capital costs* |  | | | | | | | |
| Ploughing (USD ha^–1^) | 89.38 | | | | | | | |
| *Material inputs* |  | | | | | | | |
| Seedling (USD ha^–1^) | 34 | | | | | | | |
| Urea cost (USD ha^–1^) | 0.00 | 0.00 | 16.15 | 18.36 | 24.23 | 27.53 | 32.31 | 36.71 |
| TSP cost (USD ha^–1^) | 9.05 | | | | | | | |
| MoP cost (USD ha^–1^) | 1.45 | | | | | | | |
| Gypsum cost (USD ha^–1^) | 2.07 | | | | | | | |
| ZnSO_4_ cost (USD ha^–1^) | 2.47 | | | | | | | |
| *Labor (8-hour person-days ha^-1^)* |  | | | | | | | |
| Land preparation^a^ | 2.09 | 2.09 | 2.09 | 2.09 | 2.09 | 2.09 | 2.09 | 2.09 |
| Basal fertilizer application | 1.10 | 1.10 | 1.10 | 1.10 | 1.10 | 1.10 | 1.10 | 1.10 |
| Urea fertilizer application | 0.00 | 0.00 | 1.63 | 4.50 | 1.63 | 4.75 | 1.65 | 5.02 |
| Seedling uprooting, carrying and transplanting | 31.20 | 31.20 | 31.20 | 31.20 | 31.20 | 31.20 | 31.20 | 31.20 |
| Transplanting in missing hills | 0.37 | 0.37 | 0.37 | 0.37 | 0.37 | 0.37 | 0.37 | 0.37 |
| Weeding | 9.56 | 9.56 | 11.22 | 10.76 | 13.17 | 12.21 | 14.66 | 13.67 |
| Harvesting and carrying for threshing | 23.07 | 23.02 | 23.07 | 23.02 | 22.98 | 23.02 | 23.07 | 23.07 |
| Labor wages (USD/psd) | 3.62 | | | | | | | |
| Total threshing cost (USD/ha  ) ^b^ | 33.04 | 34.10 | 39.54 | 39.81 | 36.00 | 36.07 | 31.96 | 33.34 |
| Total Variable cost (USD/ha) | 414.97 | 415.91 | 449.54 | 460.62 | 460.79 | 472.23 | 470.65 | 485.10 |
| Gross Margin (USD/ha) | 411.11 | 436.59 | 539.06 | 534.62 | 439.21 | 429.58 | 328.36 | 348.52 |

^a^ Required labor for two-wheel tractor operation and removal of rice stubble from the previous crop. Includes construction of bunds.

^b^  Crop harvest shared with thresher operators based on grain weight at 3 kg grain shared per 40 kg threshed.

**Table S5.** Mean economic inputs and outputs used to calculate profitability for trials conducted on medium-highland landscape positions in Hizla (1 USD = 82.9 Tk).

| Location | Example medium-highland trial in Hizla | | | | | | | |
| --- | --- | --- | --- | --- | --- | --- | --- | --- |
| N (Kg ha^–1^) | 0 | | 28 | | 42 | | 56 | |
| Urea Type | Prilled | USG | Prilled | USG | Prilled | USG | Prilled | USG |
| Grain yield (t ha^–1^) | 3.36 | 3.35 | 3.61 | 3.66 | 3.16 | 3.03 | 2.97 | 2.70 |
| Harvest index | 0.31 | 0.30 | 0.30 | 0.30 | 0.24 | 0.22 | 0.22 | 0.23 |
| *Capital costs* |  | | | | | | | |
| Ploughing (USD ha^–1^) | 89.68 | | | | | | | |
| *Material inputs* |  | | | | | | | |
| Seedling (USD ha^–1^) | 34.00 | | | | | | | |
| Urea cost (USD ha^–1^) | 0.00 | 0.00 | 16.15 | 18.36 | 24.23 | 27.53 | 32.31 | 36.71 |
| TSP cost (USD ha^–1^) | 9.05 | | | | | | | |
| MoP cost (USD ha^–1^) | 1.45 | | | | | | | |
| Gypsum cost (USD ha^–1^) | 2.07 | | | | | | | |
| ZnSO_4_ cost (USD ha^–1^) | 2.47 | | | | | | | |
| *Labor (8-hour person-days ha^-1^)* |  | | | | | | | |
| Land preparation^a^ | 2.15 | 2.15 | 2.15 | 2.15 | 2.15 | 2.15 | 2.15 | 2.15 |
| Basal fertilizer application | 1.15 | 1.13 | 1.12 | 1.13 | 1.12 | 1.13 | 1.13 | 1.13 |
| Urea fertilizer application | 0.00 | 0.00 | 1.67 | 4.54 | 1.68 | 4.85 | 1.69 | 5.17 |
| Seedling uprooting, carrying and transplanting | 31.20 | 31.20 | 31.20 | 31.20 | 31.20 | 31.20 | 31.20 | 31.20 |
| Transplanting in missing hills | 0.19 | 0.19 | 0.19 | 0.19 | 0.19 | 0.19 | 0.19 | 0.19 |
| Weeding | 9.48 | 9.48 | 11.04 | 10.65 | 12.81 | 12.51 | 14.08 | 13.69 |
| Harvesting and carrying for threshing | 23.02 | 22.94 | 23.07 | 22.94 | 23.11 | 22.98 | 23.11 | 23.07 |
| Labor wages (USD/psd) | 3.62 | | | | | | | |
| Total threshing cost (USD/ha  ) ^b^ | 32.42 | 32.30 | 34.82 | 35.28 | 30.51 | 29.19 | 28.71 | 26.05 |
| Total Variable cost (USD/ha) | 413.88 | 413.36 | 444.19 | 455.38 | 454.53 | 466.47 | 465.47 | 478.28 |
| Gross Margin (USD/ha) | 396.52 | 394.10 | 426.39 | 426.56 | 308.14 | 263.40 | 252.17 | 172.98 |

^a^ Required labor for two-wheel tractor operation and removal of rice stubble from the previous crop. Includes construction of bunds.

^b^  Crop harvest shared with thresher operators based on grain weight at 3 kg grain shared per 40 kg threshed.

**Table S6.** Mean economic inputs and outputs used to calculate profitability for trials conducted on medium-highland landscape positions in Mehendigonj (1 USD = 82.9 Tk).

| Location | Example medium-highland trial in Mehendigonj | | | | | | | |
| --- | --- | --- | --- | --- | --- | --- | --- | --- |
| N (Kg ha^–1^) | 0 | | 28 | | 42 | | 56 | |
| Urea Type | Prilled | USG | Prilled | USG | Prilled | USG | Prilled | USG |
| Grain yield (t ha^–1^) | 3.10 | 3.09 | 3.29 | 3.40 | 3.11 | 3.02 | 2.92 | 2.88 |
| Harvest index | 0.33 | 0.32 | 0.33 | 0.32 | 0.32 | 0.30 | 0.30 | 0.27 |
| *Capital costs* |  | | | | | | | |
| Ploughing (USD ha^–1^) | 90.67 | | | | | | | |
| *Material inputs* |  | | | | | | | |
| Seedling (USD ha^–1^) | 34.00 | | | | | | | |
| Urea cost (USD ha^–1^) | 0.00 | 0.00 | 16.15 | 18.36 | 24.23 | 27.53 | 32.31 | 36.71 |
| TSP cost (USD ha^–1^) | 9.05 | | | | | | | |
| MoP cost (USD ha^–1^) | 1.45 | | | | | | | |
| Gypsum cost (USD ha^–1^) | 2.07 | | | | | | | |
| ZnSO_4_ cost (USD ha^–1^) | 2.47 | | | | | | | |
| *Labor (8-hour person-days ha^-1^)* |  | | | | | | | |
| Land preparation^a^ | 2.17 | 2.17 | 2.17 | 2.17 | 2.17 | 2.17 | 2.17 | 2.17 |
| Basal fertilizer application | 1.12 | 1.12 | 1.12 | 1.12 | 1.12 | 1.12 | 1.12 | 1.12 |
| Urea fertilizer application | 0.00 | 0.00 | 1.67 | 4.53 | 1.67 | 4.87 | 1.67 | 5.12 |
| Seedling uprooting, carrying and transplanting | 31.15 | 31.15 | 31.15 | 31.15 | 31.15 | 31.15 | 31.15 | 31.15 |
| Transplanting in missing hills | 0.12 | 0.12 | 0.12 | 0.12 | 0.12 | 0.12 | 0.12 | 0.12 |
| Weeding | 8.49 | 8.49 | 10.11 | 9.82 | 11.81 | 11.48 | 13.20 | 12.88 |
| Harvesting and carrying for threshing | 22.87 | 22.91 | 23.00 | 23.00 | 22.96 | 22.87 | 22.96 | 22.87 |
| Labor wages (USD/psd) | 3.62 | | | | | | | |
| Total threshing cost (USD/ha  ) ^b^ | 29.92 | 29.81 | 31.78 | 32.77 | 30.04 | 29.12 | 28.15 | 27.80 |
| Total Variable cost (USD/ha) | 407.76 | 407.81 | 438.19 | 450.69 | 450.51 | 462.95 | 461.75 | 476.81 |
| Gross Margin (USD/ha) | 340.15 | 337.49 | 356.27 | 368.48 | 300.46 | 264.93 | 242.06 | 218.26 |

^a^ Required labor for two-wheel tractor operation and removal of rice stubble from the previous crop. Includes construction of bunds.

^b^  Crop harvest shared with thresher operators based on grain weight at 3 kg grain shared per 40 kg threshed.

**Table S7.** Effect of nitrogen rate and source on Agronomic Energy Input (AEI), Grain Yield Energy (GYE), Contribution of N fertilizer to AEI, Contribution of N fertilizer to GHG emissions for experiments conducted on highland landscape positions.

|  |  | AEI | GYE | Contribution of N fertilizer to AEI | Contribution of N fertilizer to GHG emissions |
| --- | --- | --- | --- | --- | --- |
| Effect | Treatment | (GJ ha^-1^) ^a^ | (GJ ha^-1^) ^a^ | (%) | (%) |
| N Source (S) | Prilled | 4.92 b | 55.39 b | 38.15 | 61.45 |
|  | USG | 4.96 a | 57.38 a | 37.88 | 61.30 |
|  |  |  |  |  |  |
| N Rate (R) | 0 | 2.63 d | 43.75 d | 0 | 0 |
| (kg N ha^-1^) | 25 | 4.20 c | 52.84 c | 36.1 | 72.7 |
|  | 50 | 5.71 b | 62.55 b | 53.2 | 84.1 |
|  | 75 | 7.24 a | 66.39 a | 62.8 | 88.75 |
|  |  |  |  |  |  |
| S × R | Prilled, 0 | 2.63 g | 44.11 f | 0 | 0 |
|  | Prilled, 25 | 4.18 f | 51.56 e | 36.2 | 72.7 |
|  | Prilled, 50 | 5.68 d | 60.82 c | 53.3 | 84.2 |
|  | Prilled, 75 | 7.20 b | 65.06 b | 63.1 | 88.9 |
|  | USG, 0 | 2.63 g | 43.39 f | 0 | 0 |
|  | USG, 25 | 4.21 e | 54.12 d | 36 | 72.6 |
|  | USG, 50 | 5.73 c | 64.29 b | 53 | 84 |
|  | USG, 75 | 7.27 a | 67.73 a | 62.5 | 88.6 |
|  |  |  |  |  |  |
| *F*-values | Source | 944*** | 79.2*** | .. | .. |
|  | Rate | 2540507*** | 498.44*** | .. | .. |
|  | S × R | 128.11*** | 20.5*** | .. | .. |

*** indicate significance at 0.001 probability.

^a^ Letters in columns not separated by blank rows indicate differences at alpha = 0.05 according to the Student’s t (for N source) or Tukey’s HSD for all other factors and interactions. LS Means separation indicated that random effects of location for Mehendigonj and Barisal Sadar were different than Hizla for AEI. LS Means separation indicated that random effects of location for Mehendigonj and Hizla were different than Barisal Sadar for GYE.

**Table S8.** Effect of nitrogen rate and source on Agronomic Energy Input (AEI), Grain Yield Energy (GYE), Contribution of N fertilizer to AEI, Contribution of N fertilizer to GHG emissions for experiments conducted on medium-highland landscape positions.

|  |  | AEI | GYE | Contribution of N fertilizer to AEI | Contribution of N fertilizer to GHG emissions |
| --- | --- | --- | --- | --- | --- |
| Effect | Treatment | (GJ ha^-1^) ^a^ | (GJ ha^-1^) ^a^ | (%) | (%) |
| N Source (S) | Prilled | 4.07 b | 50.78 | 39.65 | 64.45 |
|  | USG | 4.11 a | 50.62 | 39.32 | 64.27 |
|  |  |  |  |  |  |
| N Rate (R) | 0 | 2.14 d | 50.29 b | 0 | 0 |
| (kg N ha^-1^) | 28 | 3.88 c | 56.18 a | 43.75 | 81.25 |
|  | 42 | 4.74 b | 50.12 b | 53.7 | 86.6 |
|  | 56 | 5.61 a | 46.21 c | 60.5 | 89.6 |
|  |  |  |  |  |  |
| S × R | Prilled, 0 | 2.13 h | 50.08 | 0 | 0 |
|  | Prilled, 28 | 3.86 f | 55.73 | 43.9 | 81.4 |
|  | Prilled, 42 | 4.72 d | 50.69 | 53.9 | 86.7 |
|  | Prilled, 56 | 5.58 b | 46.63 | 60.8 | 89.7 |
|  | USG, 0 | 2.14 g | 50.51 | 0 | 0 |
|  | USG, 28 | 3.90 e | 56.63 | 43.6 | 81.1 |
|  | USG, 42 | 4.76 c | 49.55 | 53.5 | 86.5 |
|  | USG, 56 | 5.63 a | 45.78 | 60.2 | 89.5 |
|  |  |  |  |  |  |
| *F*-values | Source | 3761.2*** | 0.04 *ns* | .. | .. |
|  | Rate | 2041094*** | 18.98*** | .. | .. |
|  | S × R | 248.62*** | 0.97 *ns* | .. | .. |

*** indicate significance at 0.001 probability.

^a^ Letters in columns not separated by blank rows indicate differences at alpha = 0.05 according to the Student’s t (for N source) or Tukey’s HSD for all other factors and interactions. LS Means separation indicated that random effects of location for Mehendigonj, Barisal Sadar and Hizla were different from each other for AEI. LS Means separation indicated that random effects of location for Mehendigonj and Hizla were different than Barisal Sadar for GYE.
